# Effect of gastric cancer stem cell on gastric cancer invasion, migration and angiogenesis

**DOI:** 10.7150/ijms.46774

**Published:** 2020-07-25

**Authors:** Zhipeng Zhu, Jiuhua Xu, Lulu Li, Weipeng Ye, Guoxing Xu, Borong Chen, Junjie Zeng, Jiayi Li, Zhengjie Huang

**Affiliations:** 1 Department of Gastrointestinal Surgery, Xiamen Cancer Center, The First Affiliated Hospital of Xiamen University, Xiamen (Fujian 361003), P.R. China.; 2 Department of clinical medicine, Fujian Medical University, Fuzhou (Fujian 350004), P.R. China.; 3 Endoscopy center, The First Affiliated Hospital of Xiamen University, Xiamen (Fujian 361003), P.R. China.

**Keywords:** Gastric cancer, Cancer stem cell, Tumor angiogenesis

## Abstract

**Purpose:** Using the gastric cancer cell line SGC7901 and gastric cancer stem cell (CSC-G), we conducted this study to investigate the role of cancer stem cells in invasion, metastasis and tumor angiogenesis.

**Methods:** Stem cell markers (OCT4, SOX2, C-Myc and Klf4) expression was detected by RT-PCR and Western blotting. The proliferation, migration, invasion abilities, L-OHP and 5-FU resistance, angiogenesis were assessed using *in vitro* spherical clone formation assays, plate cloning experiments, transwell migration, transwell invasion, drug resistance, scratch-wound migration, ring formation assay, and their tumorigenic and ability were assessed using a tumor formation experiment in mice.

**Results:** Compared with the SGC7901, the expression of Oct4, Sox2, Klf4 and CD44 mRNA was significantly higher in CSC-G, the mRNA relative expression of E-cadherin in CSC-G was lower than SGC7901, while the expression of c-Myc did not significantly change. The proliferation, drug resistance, migration, and invasion abilities were significantly higher in CSC-G, and the tumorigenic ability in mice was also significantly higher.

**Conclusion:** The proliferation, drug resistance, migration, invasion, and tumorigenic abilities of CSC-G significantly were higher than SGC7901. CSC-G plays important roles in proliferation, migration, invasion, and tumorigenicity.

## Introduction

Gastric cancer is one of the most common malignant tumors in China. However, it is difficult to diagnose in early stage, with a poor prognosis. In this way, patients often do not realize until cancer progresses to middle and advanced stages. Although chemotherapy can improve the survival rate, the median survival is still less than one year 1 and the effect of molecular targeted drugs are not ideal 2. Therefore, the general treatment for advanced gastric cancer is mainly combined therapy. In recent years, some work has suggested that CSC-G is the root cause of recurrence, metastasis and drug resistance 3, 4. Therefore, the paper presents the role of CSC-G in invasion, metastasis and tumor angiogenesis.

### Formation and development of tumor stem cell theory

Before CSCs were first isolated and identified in hematological diseases in the 1960s, it was found that a small type of CSCs with self-renewal and differentiation capacity exists within various types of solid cancers, such as colon cancer, breast cancer, liver cancer, lung cancer and pancreatic cancer 4. This capacity has also been validated, including self-renewal, efficient DNA repair and multilineage differentiation; even very few CSCs could significantly promote cancer growth, recurrence and metastasis 5. CSCs could maintain self-renewal through asymmetric division, and proliferation and infiltration through symmetrical division 6. Previous studies have shown that CSCs enriched after neoadjuvant chemotherapy or conventional treatment, which may be the main cause of recurrence 6, 7. Therefore, with respect to the specific biological characteristics of CSCs, we should incline to develop more specific molecular targeted drugs for the treatment of gastric cancer.

As the start of cellular reprogramming in 2006, Yamanaka induced fibroblasts to reprogram and differentiate into pluripotent embryonic stem cells via OCT4, SOX2, C-Myc and Klf4 8. With the research furtherly developed, reprogrammed cell is not limited to specific cell type and specific differentiation stage, somatic cell from different tissue sources could be reprogrammed into corresponding pluripotent stem cells or corresponding disease cell models, such as gastrointestinal epithelial cells and somatic cell from different disease models 8. At present, OCT4, SOX2, C-Myc and KLF4 are considered as the core transcription factors that maintain the self-renewal capacity, and highly expressed in a variety of cancers, such as bladder cancer, prostate cancer, chronic myeloid leukemia, glioma, orthotopic colon cancer, lung adenocarcinoma, and pancreatic cancer 9-11. Domestic and foreign researches have shown that several types of CSCs have significant differentiation potential, and could form endothelium and complex 3D glandular structures *in vitro* 6, such as in embryonic stem cells and CSC-G.

### Development of tumor angiogenesis theory

In the early stage, cancer cells could get nutrition to sustain growth mainly through tissue infiltration, if the nutrition could not be provided by neovascularization, cancer cells will be in a long-term inhibition state 12, 13. Therefore, angiogenesis is an important part of the therapeutic regimen. From professor Folkman putting forward the angiogenesis theory in the 1970s to the first anti-tumor angiogenic molecular targeted drug approved by the FDA, angiogenesis mechanism has been studied and perfected for more than 30 years 14. It is acknowledged that fresh capillary is generated from existing capillaries by means of “sprouting” 14. Nevertheless, the latest experimental evidence suggests that CSCs differentiate into vascular endothelium to participate the angiogenesis. The capacity of CSCs differentiating blood vessels was first derived from the study of glioma CSCs, and CD133+ CSCs could directly differentiate into vascular endothelial cell *in vivo* and *in vitro* 15. Some studies have shown that bevacizumab could reduce the blood supply, meanwhile, it could also induce and accelerate invasion, recurrence and metastasis; the main reason being that vascular endothelial cells are derived from the CSCs, which can resist vascular molecule target drugs 16.

CSCs and differentiated vascular endothelium are not sensitive to current conventional chemotherapy and molecular targeted drugs. What is worse, molecular targeted therapy ostensibly reduces the blood supply of the tumor, but accelerates tumor invasion and metastasis, which is the main reason of failure of molecular targeted therapy in clinic. Therefore, it is of great significance to deeply study the biological characteristics of cancer stem cell and the mechanism of vascular differentiation.

## Methods

### Materials

#### Drugs and reagent

We obtained basic medium (Hyclone); ordinary fetal calf serum (PAA); 0.25% trypsin, DMEM/F12 medium, 50xb27 supplement, 100xn2 supplement, EGF and bFGF (Gibco); cell lysis solution Trizol (Life); PrimeScript™ RT-PCR Kit, Maxima SYBR Green/ROX qPCR Master Mix (2×) (Dalian Takara co., LTD); anti-OCT4 antibody produced in rabbit, anti-SOX2 antibody produced in rabbit, anti - Epcam antibody produced in rabbit(Abcam); goat anti-mouse/rabbit double-antibody purchased from BOSTER company, Peroxidase conjugate-goat anti-mouse poly (Sigma); Real-time fluorescence quantitative PCR primers (Invitrogen company).

#### Cell line

Human gastric cancer SGC7901 cell lines were provided by the cancer center of the First Affiliated Hospital of Xiamen University, while CSC-G cell lines were provided by the digestive center, Xijin Hospital, The fourth Military medical university.

#### Animals

10 NOD/SCID female mice (4 weeks, 3.2 g) were purchased from Xiamen University Laboratory Animal Center.

### *In vitro* experiment

#### Cell culture

SGC7901 were cultured with RPMI1640 medium containing 10% FBS at 37°C in a saturated humidified atmosphere containing 5% CO2, fresh media was changed every 2-3 days. The cell cultures were maintained in monolayer and passaged when they reached 90%confluence; then cells were digested with 0.25% trypsin, which was removed after dilatation of the intercellular spaces was observed. CSC-G were suspension-growing cells, cultivated in ultra-low adhesion culture dish with serum-free medium at 37°C in a saturated humidified atmosphere containing 5% CO^2^, And the serum-free medium was comprised of DMEM/F12 (1:1), B27 (2%), EGF (20 ng/ml) and bFGF (20 ng/ml), moderate medium was added every day after inoculation, and CSC-G passaged for 7-10 days.

### Extraction of total RNA and RT-PCR

RNA was extracted and reverse transcription was conducted respectively according to the operation scheme of TRNzol total RNA extraction Kit and PrimeScript ™ RT-PCR Kit. The primer upstream sequence of OCT-4 was CCTGAAGCAGAA GAGGATC and the primer downstream sequence was CGTTTGGCTGAAT ACCTT. The primer upstream sequence of SOX2 primer was CCAGCTCGCAG ACCTACAT and the primer downstream sequence was ACTTGACCACCGA ACCCA. The primer upstream sequence of C-Myc is CACCAGCAGCGACTCTGA and the primer downstream sequence is GATCCAGACTCTGACCTTTTGC. The primer downstream sequence of Klf4 is ATTGGACCCGGTGTACATTC and the primer downstream sequence is AGCACGAACTTGCCCATC. The primer upstream sequence of E-cadherin is ATCGTCAATGCCAG TGTAC and the primer downstream sequence is CTGCCTTCATCACCAAAC. The primer upstream sequence of CD44 primer is CAAGCAATAGGA ATGATGTC and the primer downstream sequence is GGTCACTGGGA TGAAGGT. The primer upstream sequence of GAPDH was GCACCGTCAAGGCTGAGAAC and the primer downstream sequence was TGGTGAAGACGCCAGTGGA. Real Time-PCR amplification conditions consisted of initial denaturizing step at 95°C (30 s) followed by 45 cycles of step protocol consisting of 95°C (15 s), 58°C (15 s), 72°C (20 s). Finally, ROCHE Light cycler 480 built-in software was used to analyze experimental data and relative mRNA expression was calculated using ^2-ΔΔCt^ method.

### Western blotting

Cell proteins were extracted using RIPA lysis buffer. Western blotting was performed using the standard procedure. The primary antibodies were anti-OCT4, anti-SOX2 and anti-Epcam antibodies. Goat anti-mouse/rabbit double antibodies were used as secondary antibodies. The enhanced chemiluminescence (ECL) was used for coloration, radiography and observation.

### Immunohistochemical detection

Paraffin embedded cell slide and tissue slice were were dewaxed to water (the cell slide were hydrated), washed with PBS for 3 times with 5 minutes each time. Next, the antigen was repaired by 0.01 M sodium citrate buffer solution (pH 6.0) with water -bath heating to about 95°C for 15 minutes. Then, the antigen was sealed at room temperature with goat serum sealant for 30 minutes, and the excess sealant was removed. Furthermore, anti-incubation tissue slices (cell creeping slices) were taken out by overnight dripping of anti-50 ul anti-incubation. When temperature went room temperature for 30 minutes, the PBS was used for washing three times with 5 minutes each time. 50 ul of bivalent antibody was added and was incubated at room temperature for 30 minutes. Additionally, PBS was used to wash for three times, DAB was used to develop color at room temperature for 3-7 minutes, distilled water was used to wash and hematoxylin was used for re-dying for 1-3 minutes, sealed using gum after dehydration and observed under microscope.

### Spherical clone formation experiment

3×10^3^ SGC7901 and CSC-G cells were inoculated into the ultra-low adhesion 6-well plates, and cultured in 3 ml serum-free medium consisting of DMEM/F12 (1:1), B27 (2%), EGF (20 ng/ml) and bFGF (20 ng/ml). The formation of spherical clones was measured every day.

### Plate cloning assay

3×10^3^ SGC7901 and CSC-G cells were inoculated in 6-well plates, and cultured in 3 ml RPMI-1640 (10% FBS) culture medium was added into 6-well plates after culturing for 10 days. 5 fields (×40 times) were randomly selected to count the number of each field.

### Transwell assay to detect invasiveness

First, 100 u1 serum-free culture medium was added to the Transwell upper chamber, while 600 u1 culture medium containing 20% fetal calf serum was added to the lower chamber. Next, 200 u1 SGC7901 and CSC-G cell suspension was added to the upper chamber respectively (2×10^5^ /ml). After 20-24 hours culture, the cells were fixed, stained, dried, and counted.

### Transwell assay to assess migration

The cells were starved for 12-24 hours. Culture medium containing 600 µL 20% serum concentration was then added into every well of a 24-well plate. Next, 200 µL cell solution (2×10^5^/mL) was added into the transwell chamber, and the chamber was placed into the transwell well. After culturing in an incubator, we calculated the number of cells that penetrated the membrane at different time points, wiped off the cells in the inner chamber using a cotton swab, fixed the migrated cells using methanol, stained the cells using crystal violet, and observed the semipermeable membrane using a microscope.

### Scratch-wound migration assay

Cells were plated on a six-well plate (5×10^5^ cells/mL). After the cells spread over the plate, a pipette was used to draw a horizontal line at the back of the six-well plate, then the plate was washed with PBS and placed in an incubator containing serum-free RPMI 1640 medium for 24 hours. An optical microscope was used to observe migration at 0 and 24 hours, and the results were photographed. ImageJ software was used to measure the width of the scratch area at 0 and 10 hours. The migration speed was quantitatively compared by calculating the difference in the distance between the two edges of the scratch between 0 and 10 hours.

### Ring formation assay

The matrigel matrix was diluted 1:3 with serum-free matrix (on the ice). Next, matrix was dispensed in 96-well plates evenly, after culturing in an incubator at 37°C for half an hour, air drying for standby application. The cells within logarithmic growth phase were starved for 12-24 hours and adjusted the cell density of 2×10^5^. Finally we added the adjusted cell liquid to air-dried matrix, observed the number of cyclization after culturing at 37°C for 8 hours.

### MTS test to assess the drug inhibition rate

Cells in the logarithmic growth stage (5×10^4^/mL) were inoculated onto a 96-well plate, and 200 µL oxaliplatin (L-OHP; 0.1, 0.2, 0.4, 0.8, 1.6, 3.2, or 6.4 µg/mL) or fluorouracil (5-FU; 1, 2, 4, 8, 16, 32, or 6.4 µg/mL) diluted with culture medium was added (three wells for each concentration). The control group was adding cell suspension only. When the time point was reached, 20 µL MTS was added into each well to culture at 37°C for 2.5 hrs. OD 490 nm was assessed using an enzyme-linked immune detector to calculate the cell inhibition rate according to the following formula:

Cell inhibition rate (%) =1 - (OD_experimental_/OD_control_) ×100%

### *In vivo* experiment

#### Tumor formation experiment in nude mice

10 NOD/SCID mice were randomly divided into two groups with 5/group. According to the random number table, 1×10^6^ SGC7901 cells and CSC-G suspension cells were injected subcutaneously in the right back of each nude mouse. During the two weeks, Tumor volume was monitored and calculated according to the formula:

*V* = 0.526 × *L* (length) × *W^2^* (width)

by measuring tumor length and width every 2 days. At the end of second week, each mouse was euthanized (by cervical dislocation), and the tumor was removed for weighing.

### Statistical Analysis

Statistical analyses were performed using SPSS 16.0 for windows (SPSS 16, Chicago, IL). Continuous numerical data were expressed as median and interquartile range or mean and standard deviation. *P*<0.05 was considered statistically significant.

## Results

### Culture of gastric cancer stem cells

**Figure 1:**
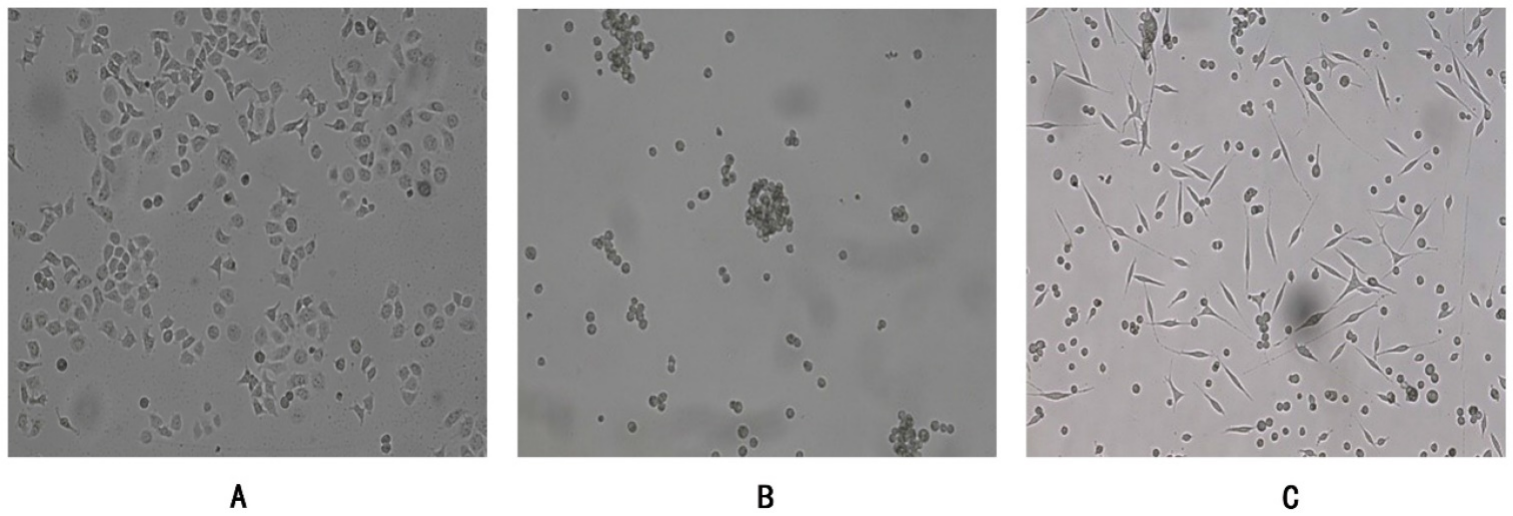
SGC7901 and CSC-G (x 100 times) were grown under different culture conditions. (**A**) Adherent growth of SGC7901 (**B**) Suspension growth of CSC-G (**C**) Adherent growth of CSC-G.

Human gastric cancer cells SGC7901 were adherent growth cells, the culture condition was RPMI-1640 (10%FBS), CSC-G were suspension-growing cells, the culture condition was DMEM/F12 (1:1) serum-free culture medium containing EGF (20 ng/ml) and bFGF (20 ng/ml), and the adherent CSC-G were in the mesenchymal shape (**Figure 1**). The culture atmosphere was maintained at 37°C in a saturated humidified atmosphere containing 5% CO m^2^.

### Comparison of the expression of mRNA and proteins

#### Real-time PCR detecting the relative mRNA expression levels of marker genes

**Figure 2:**
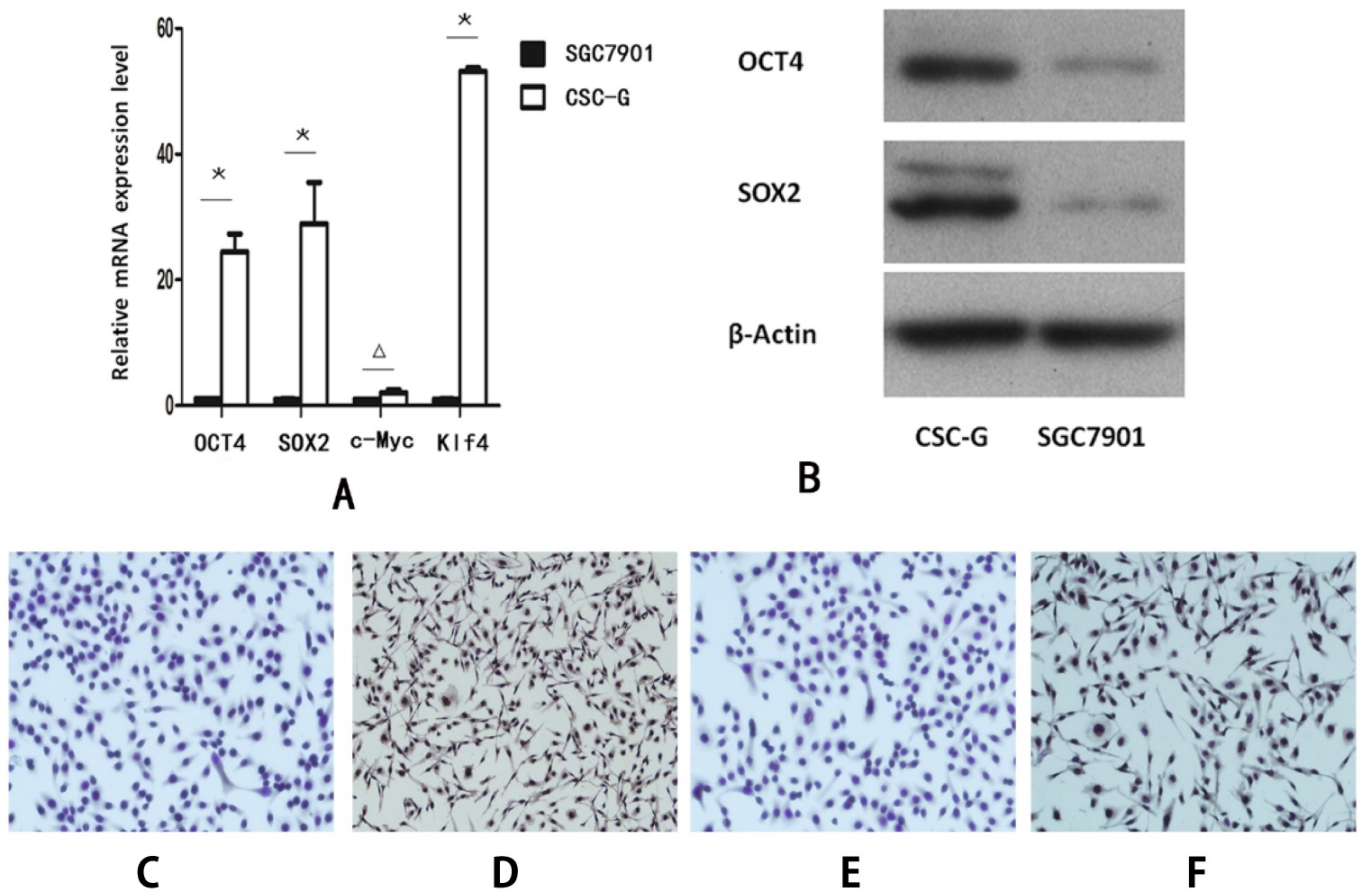
The expression level of stem cell marker genes in CSC-G and SGC7901. (**A**) The relative expression of mRNA in OCT4, SOX2, c-Myc and Klf4 (**B**) The expression level of proteins in OCT4 and SOX2 (**C**) OCT4 expression in SGC7901 (x 100 times) (**D**) OCT4 expression in CSC-G (x 100 times) (**E**) SOX2 expression in SGC7901 (x 100 times) (**F**) SOX2 expression in CSC-G (x 100 times).

Compared with SGC7901, the relative mRNA expression level of OCT4, SOX2 and Klf4 in CSC-G was significantly higher (*P*<0.05). However, there was no significant difference in the mRNA expression level of C-Myc (*P*>0.05) (**Figure 2A**).

#### Western blotting detecting the expression levels of OCT4 and SOX2 proteins

Compared with SGC7901, The protein expression level of both OCT4 and SOX2 in CSC-G was higher (*P*<0.05) (**Figure 2B**).

#### Cellular immunohistochemistry revealed the positive rate of OCT4 and SOX2

The protein expression level of OCT4 and SOX2 was detected by cellular immunohistochemistry. CSC-G had stronger OCT4 staining than SGC7901 (**Figure 2C-D**) and SOX2 presented stronger staining in CSC-G than SGC7901 (**Figure 2E,F**).

### Comparison of the biological characteristics of gastric cancer stem cells and gastric cancer cells

#### Spherical cloning assay

**Figure 3:**
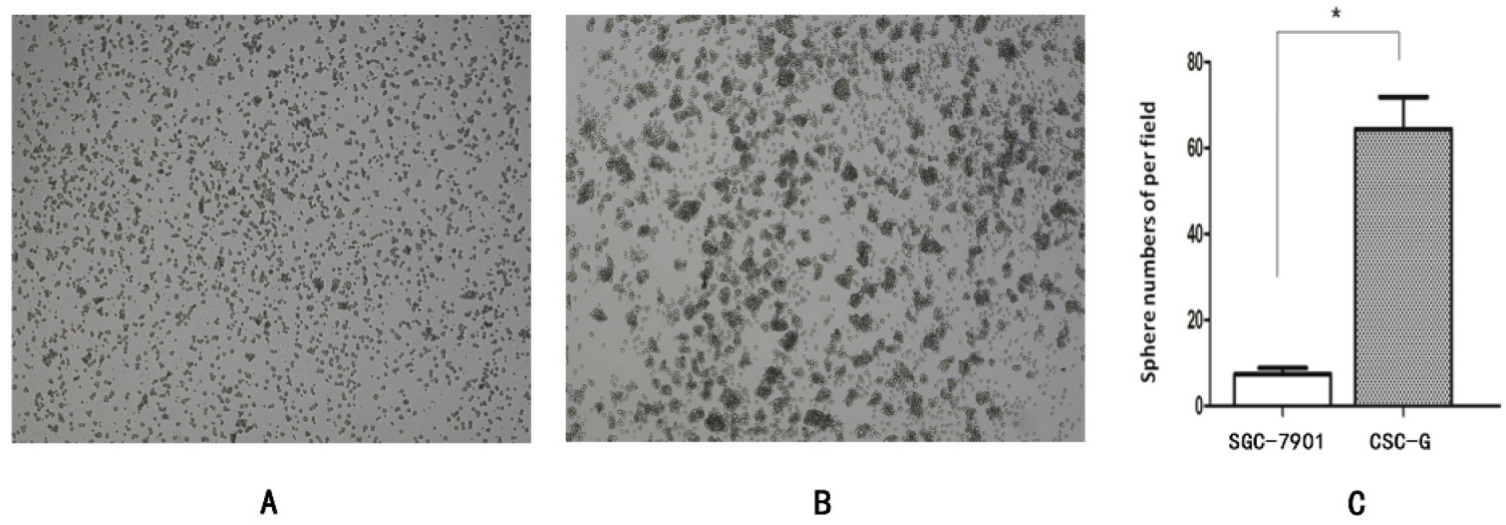
Tumor cell ball image that cells in each group were suspension cultured for 14 days (x 40 times). (**A**) SGC7901 (**B**) CSC-G (**C**) Tumor cell ball bar graph.

The number of CSC-G and SGC7901 exhibiting spherical formation was 64.33 ± 7.51 and 6.50 ± 0.71. Compared with SGC7901, the tumorigenic ability of CSC-G was significantly stronger (*P*<0.05) (**Figure 3**).

#### Plate clone formation assay

**Figure 4:**
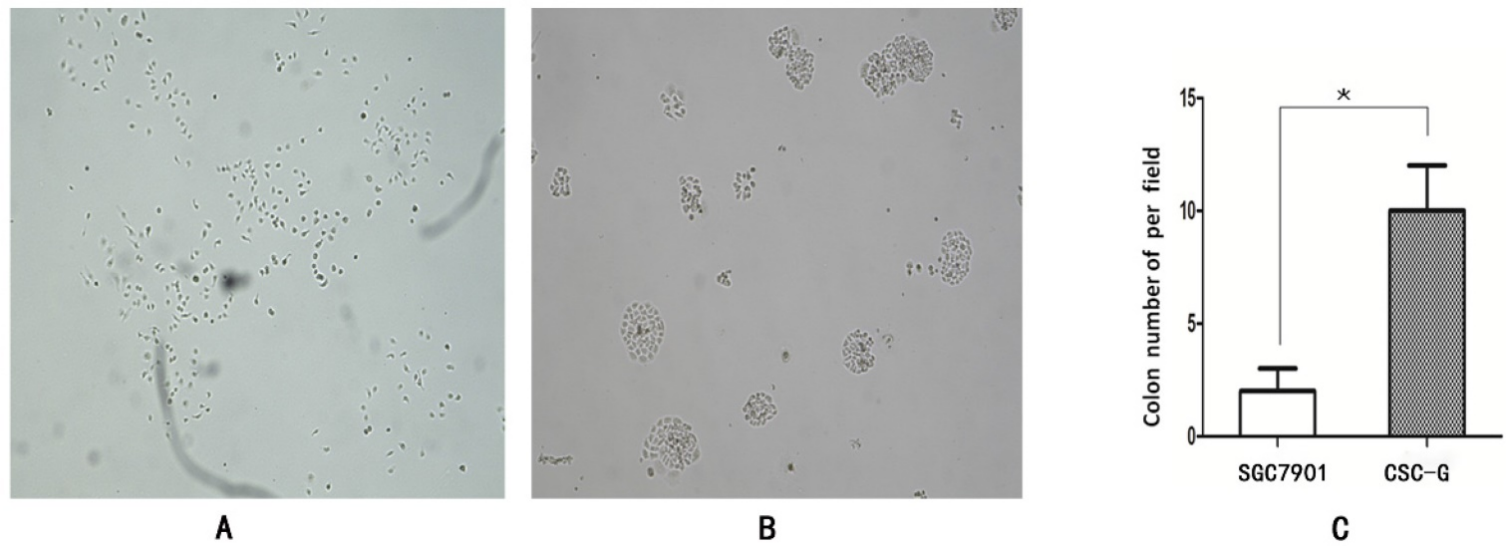
The formation of plate clone in each group (x 40 times) (**A**) SGC7901 (**B**) CSC-G (**C**) Tumor cell ball bar graph.

The number of CSC-G and SGC7901 exhibiting cell clones formation was 9.60 ± 1.67 and 1.80 ± 0.84. Compared with SGC7901, the tumorigenic ability of CSC-G was significantly stronger (*P*<0.05) (**Figure 4**).

#### Scratch-wound migration assay

**Figure 5:**
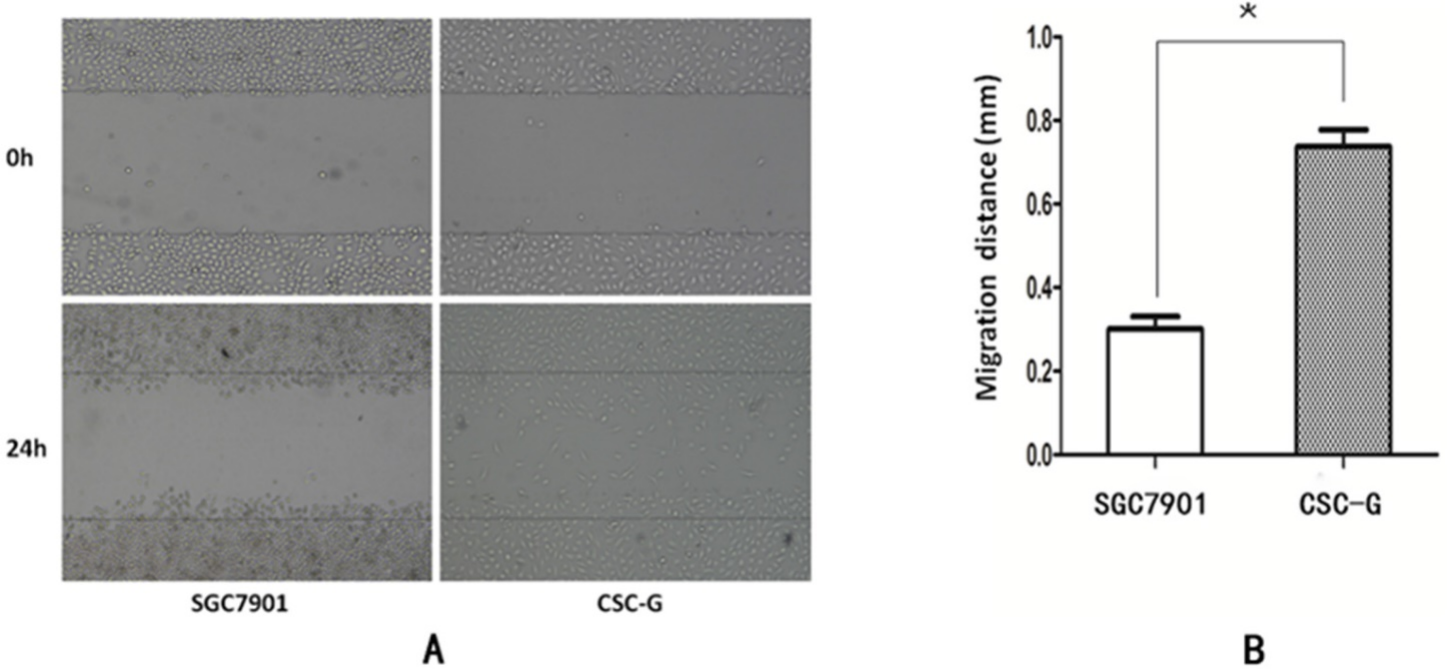
(**A**) The cell migration image of the 0th and the 24th (**B**) Comparison of the migration distance in SGC7901 and CSC-G.

The width was observed under the microscope at 0h and 24h after scratching. The relative migration distance of CSC-G and SGC7901 was 0.75 ± 0.023 and 0.31 ± 0.016, respectively. Compared with SGC7901, the migration ability of CSC-G was significantly higher (*P*<0.05) (**Figure 5**).

#### Transwell migration assay

**Figure 6:**
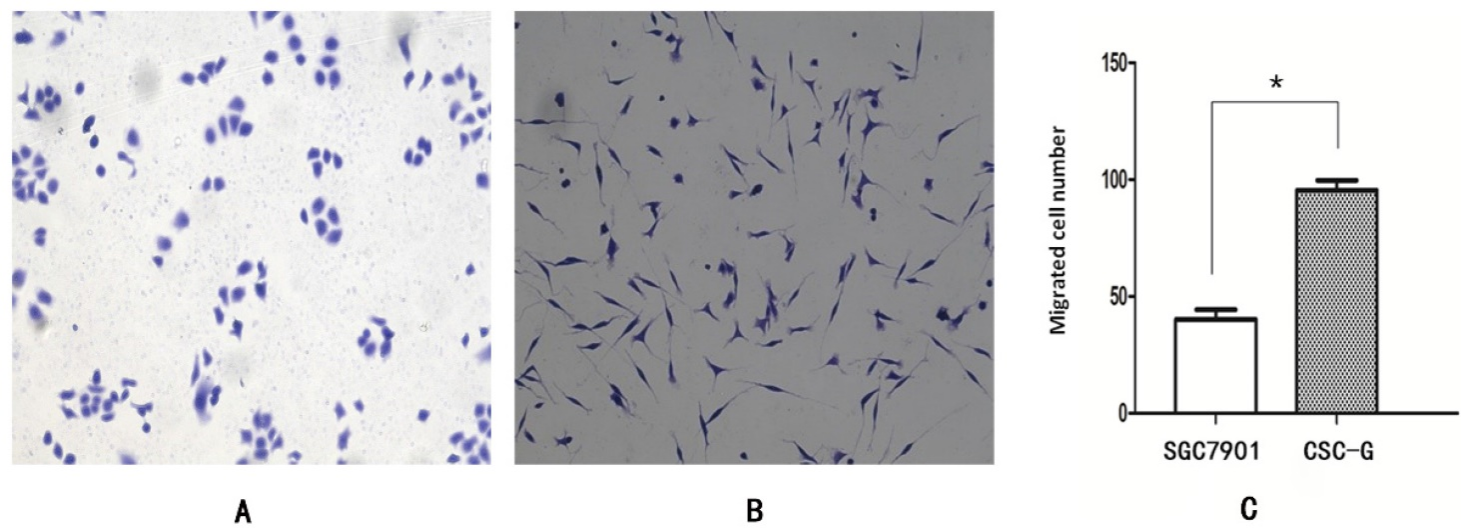
The migration capacity of different cells was compared with 0.1% crystal violet staining (x 100 times). (**A**) SGC7901 (**B**) CSC-G (**C**) The number of cells in each cell passing through the basement membrane in the migration experiment.

The mean number of SGC7901 and CSC-G cells passing through the basement membrane was 40.20 ± 4.15 and 97.60 ± 3.58, respectively. Compared with SGC7901, the migration ability of CSC-G was significantly increased (*P*<0.05), which indicated CSC-G have greater migration ability (**Figure 6**).

#### Transwell invasion assay

**Figure 7:**
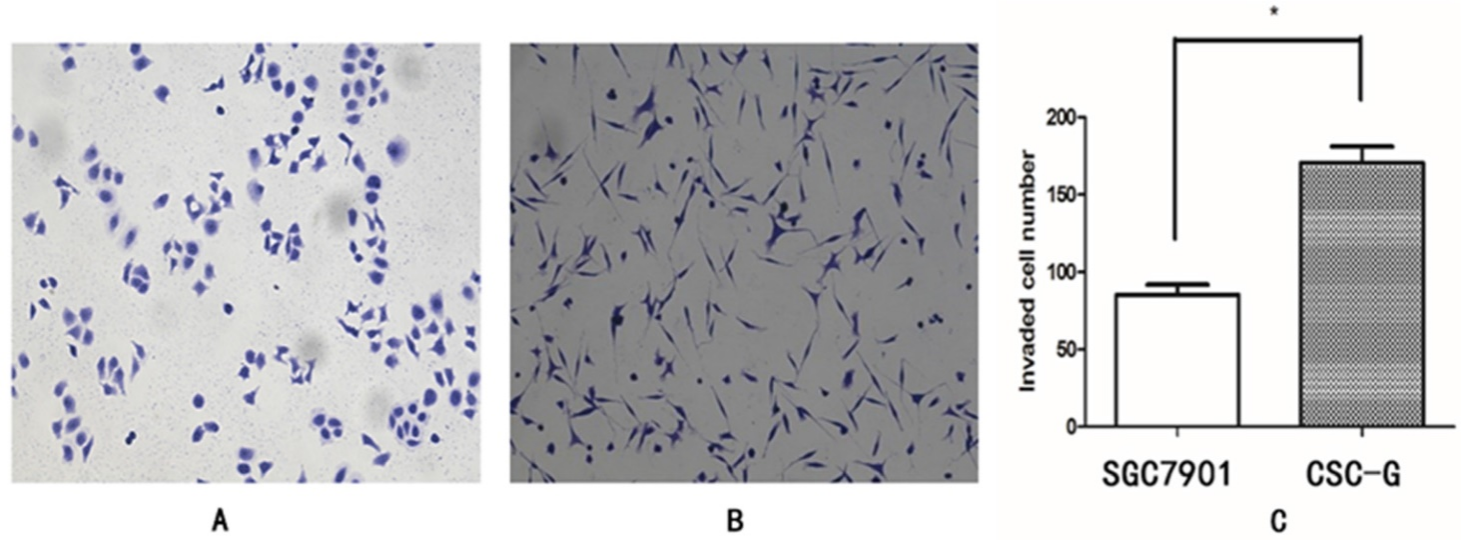
Comparison of cell invasiveness, 0.1% crystal violet staining (x 100 times). (**A**)SGC7901 (**B**) CSC-G (**C**) The number of cells in each cell passing through the basement membrane in the invasive experiment.

The mean number of SGC7901 and CSC-G cells invading and passing through the basement membrane was 85.20 ± 6.57 and 170.60 ± 10.43, respectively. This indicated that CSC-G cells led to greater invasion ability (*P*< 0.05) (**Figure 7**).

#### Real-time PCR detecting the relative mRNA expression level of CD44 and E-cadherin

**Figure 8:**
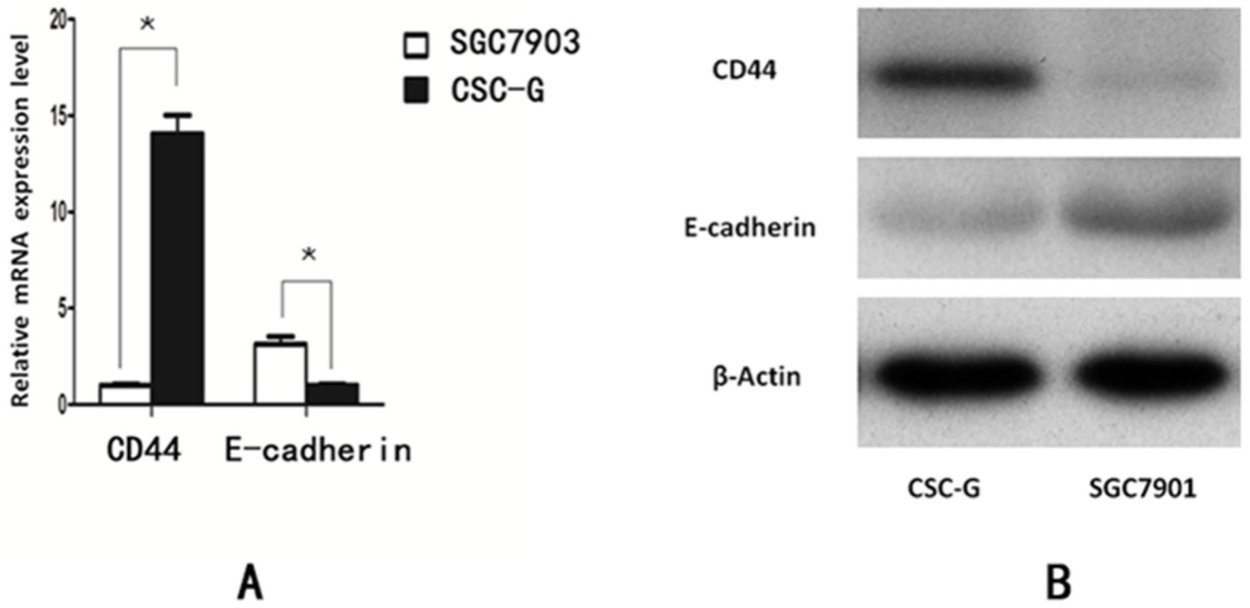
The expression level of stem cell marker in CSC-G and SGC7901. (**A**) Expression of mRNA in CD44 and E-cadherin (**B**) Expression of protein in CD44 and E-cadherin.

Compared with SGC7901, The relative mRNA expression of CD44 in CSC-G was higher (*P*<0.05). The relative mRNA expression of E-cadherin in CSC-G was significantly lower (*P*<0.05). The expression level of E-cadherin and CD44 detected by Western blotting was consistent with that detected by Real-time PCR (*P*<0.05) (**Figure 8**).

#### MTS assay to detect drug resistance

**Figure 9:**
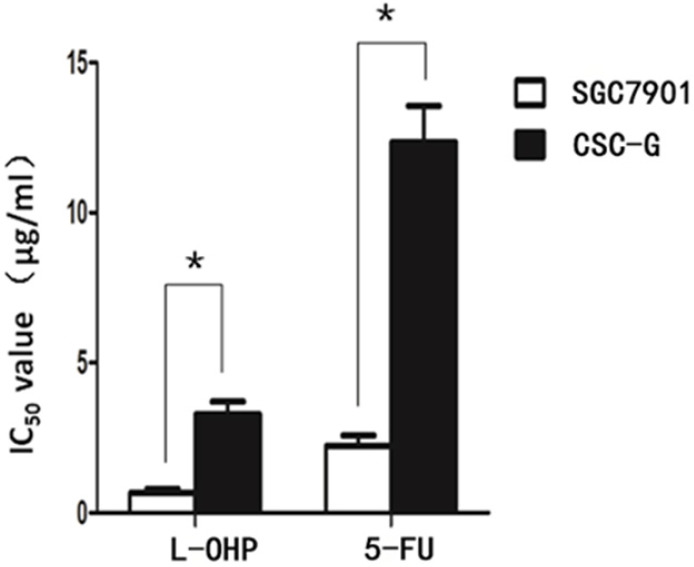
IC50 of SGC7901 and CSC-G cells influenced by L-OHP and 5-FU.

After L-OHP was administered to the two groups, the half maximal inhibitory concentration (IC50) of SGC7901 and CSC-G was 0.67 ug/ml and 3.28 ug/ml, respectively. After 5-FU was administered to the two groups, the IC50 of SGC7901 and CSC-G was 2.23 ug/ml and 12.34 ug/ml, respectively. Compared with SGC7901, CSC-G had significantly higher L-OHP and 5-FU resistance (*P*<0.05). This indicated that CSC-G led to stronger drug resistance (**Figure 9**).

#### Tumor formation experiment in nude mice

**Figure 10:**
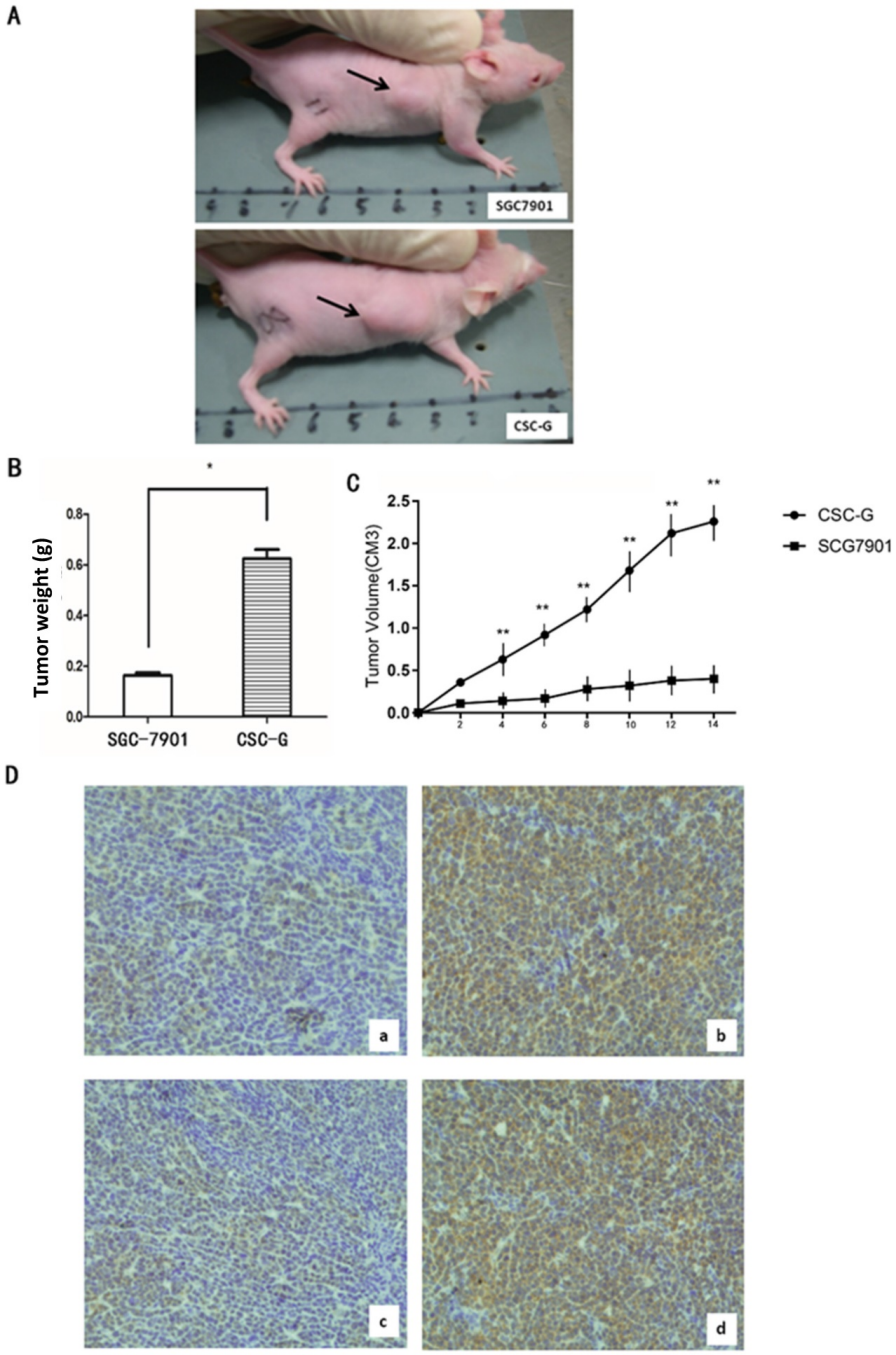
(**A**) The transplanted tumor of two types of cells in nude mice; (**B**) Final tumor weight of transplanted tumor (g); (**C**) Mice tumor volume curves (**D**) Immunohistochemistry: OCT4 expression in (a)SGC7901 and (b) CSC-G; SOX2 expression in (c) SGC7901and (d) CSC-G.

The final weight of transplanted tumor of CSC-G and SGC7901 was (0.624 ± 0.036) and (0.164 ± 0.010), respectively. Compared with SGC7901, the weight for CSC-G was significantly heavier (*P*<0.05). Tumor volume measurements in mice demonstrate that SGC7901 showed a slower increase in tumor volume compared with the CSC-G. This showed that overexpression of CSC-G led to a greater tumorigenic ability (**Figure 10A-C**).

#### Immunohistochemistry detecting the expression levels of OCT4 and SOX2

Tumor tissue was taken out from nude mice for histology, and the protein expression level of OCT4 and SOX2 was detected by immunohistochemistry. CSC-G had stronger OCT4 and SOX2 staining than SGC7901 (**Figure 10D**).

### Role of gastric cancer stem cells in cancer angiogenesis

#### Ring formation experiments

**Figure 11:**
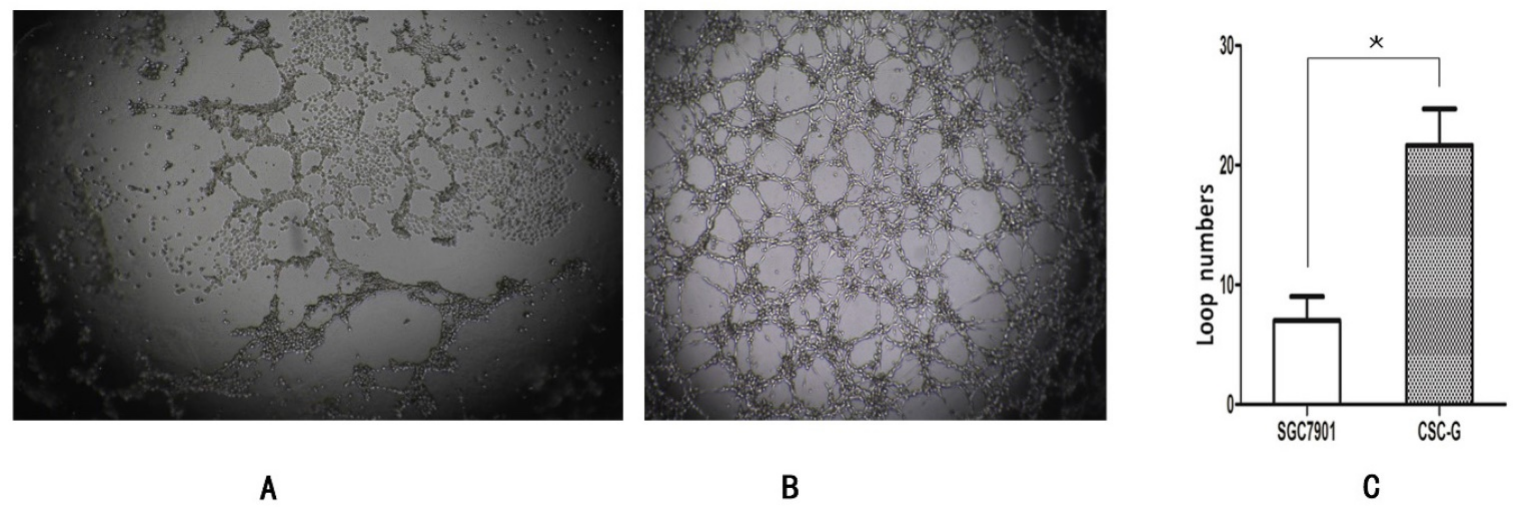
The ring forming capacity of two types of cells. (**A**) Ring diagram of SGC7901 group. (**B**) Ring diagram of CSC-G. (**C**) The number of ring in the two groups.

The number of CSC-G and SGC7901 exhibiting cyclization was 19.0 ± 6.52 and 7.20 ± 1.48. Compared with SGC7901, the tumorigenic ability of CSC-G was significantly stronger (*P*<0.05) (**Figure 11**).

## Discussion

In recent years, great progress has been achieved in gastric cancer, but the pathogenesis is still not very clear. With the deepening the relationship between cancer and stem cell, more and more people believe that cancer is a type of stem cell disease 17, which opens a new field for the research and treatment of gastric cancer. The concept of CSC had been put forward as early as one century ago. In 2001, Reya et al. supplemented and enriched the theory of CSC through the study of hematologic cancer and hematopoietic stem cell 18. In 2006, American Association for Cancer Research (AACR) defined cancer stem cells as a small group of tumor cells with self-renewal and multiple differentiation potential 18. In recent years, many reports indicated the CSC in various types of solid tumors, such as brain tumor, breast cancer, colon cancer, prostate cancer and liver cancer 19-25. However, there are relatively few reports related to gastric cancer stem cell. Until 2009, Takaishi et al first isolated cancer cells with stem cell characteristics in multiple gastric cancer cell lines 26. Many cancer arise from mutations of the adult cells, but the gastrointestinal epithelium updated quickly, it is difficult to cause malignant lesions, while the mutation of stem cell with a very long lifespan is more likely to accumulate and eventually cause cancer, so it is widely believed that gastric cancer arises from the mutation of gastric adult stem cell 27.

In this study, we found that the relative mRNA expression level of CD44, OCT4, SOX2 and Klf4 were significantly higher in CSC-G than SGC7901, and the tumorigenicity and invasiveness of CSC-G were significantly higher than SGC790. At present, there are many ways to isolate and identify gastric cancer stem cell, and cell surface markers are the most important for isolating and identifying gastric cancer stem cell. Nishii 28 found that high expression of stem cell markers could isolate gastric cancer cell line with high potential for peritoneal metastasis, such as CD44, OCT4 and SOX2. Schmuck et al. 29 were able to select cell with high expression of CD133 from AGS and MKN45 gastric cancer cell. *In vitro* experiments, Takaishi 26 added the selected CD44 positive gastric cancer cell into the serum-free medium containing EGF and bFGF, and they could form spherical clones after a few weeks. Meanwhile, they implanted a few gastric cancer cells into the skin of the immunodeficient mice for several weeks, which showed a strong tumorigenic ability. Therefore, CD44, CD133, OCT4 and SOX2 are generally considered as the surface markers of gastric cancer stem cell. And we believe that the high expression of stem cell markers is the cause of recurrence and metastasis of gastric cancer.

The study also found the expression level of both OCT4 and SOX2 protein in CSC-G group was higher than those in SGC7901. Matsuoka 30 analyzed the expression levels of OCT4 and SOX2 in gastric cancer specimens by immunohistochemistry, and found that positive expressions of SOX2 and OCT4 in gastric cancer tissues were associated with gastric cancer invasion, may be independent factors affecting the prognosis. Yang 31 screened out CSC-G cells from SGC7901 cell line using serum-free medium, CSC-G cells were highly tumorigenic in nude mice and highly expressed stem cell markers OCT4 and SOX2. Wang 32 found that abnormal expression of CD44 in gastric cancer tissues suggesting a poorer prognosis, this was consistent with our research.

Furthermore, we found that the mRNA expression level of E-cadherin in CSC-G cells was down-regulated compared with SGC7901, the relative mRNA expression of CD44 in CSC-G group was higher than that in SGC7901 group. E-cadherin is an important adhesion molecule, when the expression is down-regulated or dysfunction, the cells will lose adhesion capacity, which means that cancer cells are highly invasive with metastasizing to local lymph nodes or distant metastases easily 33. It has previously been shown that down-regulated expression of E-cadherin is positively correlated with invasion and metastasis ability of gastric cancer stem cells 31. Therefore, gastric cancer stem cells are more aggressive and metastatic than common gastric cancer cells. Takaishi proved that the CD44 (+) gastric cancer stem cells were more resistant to 5-fluorouracil (5-FU), etoposide (VP-16) and radiation than the CD44 (-) gastric cancer cells 26. Our study found that the IC50 of chemotherapeutic drugs oxaliplatin (L-OHP) and 5-fluorouracil (5-FU) for CSC-G was significantly higher than that for SGC7901, which suggested that the gastric cancer stem cells may be one of the mechanisms of chemotherapy resistance.

In our study, we found that CSC-G has stronger migration ability compared with SGC7901, and could form vascular loop structure *in vitro*, which suggested that CSC-G played a vital role in the angiogenesis. Recent studies have concluded that there is a strong relationship between CSC and cancer angiogenesis, so anti-cancer angiogenesis therapy is difficult to achieve the desired effect 34. Cancer angiogenesis is essential for growth, formation and maintenance 35. Compared with cancer cell, CSC showed stronger tumorigenesis and formation ability, not only CSC have self-renewal and proliferation ability, but also could promote tumor angiogenesis 36. CSC promote cancer angiogenesis via several mechanisms include: over-expression of several angiogenic factors, such as VEGF, angiopoietin, EGF. Some recruiting host cells could secrete angiogenic factors including macrophages, lymphocytes, fibroblasts 37. Extracellular matrix and cancer stem cells could secrete proteins and enzymes to promote angiogenesis; CSC could differentiate into cancer vascular progenitor cells and endothelial cells, which are directly involved in the angiogenesis or the formation of tubule structures without endothelium to participate in the cancer microcirculation 38, 39.

Conventional anticancer therapy is mainly to kill differentiated and highly proliferative cells, while CSC is in the resting stage of proliferation, poorly differentiated or undifferentiated stage, which could escape chemotherapy, initiate and maintain the subsequent progress of tumors via anti-apoptosis mechanism. Overall, we may conclude that the invasiveness, tumorigenicity, drug resistance migration and angiogenesis capability of gastric cancer cell is significantly higher than cancer cell, as well as the expression of several markers, thus promising a new target for managing gastric cancer, we could study some targeted molecular drugs targeting to marker gene such as CD44, OCT4, SOX2 and Klf4, and we could also dedicate to study some drugs to inhibit cancer angiogenesis to eliminate the gastric cancer. However, several limitations to the study should be acknowledged. First, multiple types of GC cells should be performed to prove our point. Second, more NOD/SCID mice could be used to increase reliability of our study. Third, further biological experiments should be performed to reveal the underlying mechanism. Fourth, it failed to identify the underlying mechanism as to how CD44, CD133, OCT4, SOX2 take a part in the recurrence and metastasis of gastric cancer. Therefore, more studies urgently need to be carried out so as to benefit people with gastric cancer.

## Conclusion

We may conclude that the invasiveness, tumorigenicity, drug resistance migration and angiogenesis capability of CSC-G were significantly higher than cancer cell, as well as the expression of marker genes. Therefore, the existence of CSC-G might be a mechanism of gastric cancer recurrence, metastasis, and drug resistance.
